# Hyaluronate-Alginate Gel-Coated Porcine Small Intestine Submucosa for Nerve Protection Minimizes Extraneural Collagen Deposition in a Preclinical Model

**DOI:** 10.1016/j.jhsg.2025.100784

**Published:** 2025-07-29

**Authors:** Nesreen Zoghoul Alsmadi, Curt Deister, Peter Evans, Tamer Ghanem, Brandon Smetana, Deana Mercer

**Affiliations:** ∗ Axogen Corporation, R&D, Tampa, FL; † Cleveland Clinic Martin Health, Orthopaedic Surgery, Stuart, FL; ‡ Premier Head and Neck Surgery, Flint, MI; § Indiana Hand to Shoulder Center, Indianapolis, IN; ‖ The University of New Mexico, UNM Orthopedics & Rehabilitation, Albuquerque, NM

**Keywords:** Nerve adhesion, Nerve wrap, Peripheral nerve repair, Preclinical nerve, SIS

## Abstract

**Purpose:**

This study evaluated the use of a hyaluronate-alginate gel-coated small intestine submucosa (HA-SIS) to protect a nontransected nerve in an adhesion rodent model.

**Methods:**

The sciatic nerve of 60 male Lewis rats underwent neurolysis, with subsequent surgical treatments varying according to their assigned groups. The sham group (n = 20) was surgically closed immediately after neurolysis. The muscle bed of the untreated group (n = 20) was traumatized with a bipolar coagulator and then surgically closed. The muscle bed of the HA-SIS group (n = 20) was traumatized with a bipolar coagulator, the nerve was wrapped with HA-SIS, and the site was surgically closed. Ten animals in each group were terminated at each timepoint, 6 and 26 weeks. Surgical sites were assessed by gross pathology and histology.

**Results:**

Gastrocnemius muscle wet weight was significantly higher in the HA-SIS group than in the untreated and sham groups at 26 weeks. At both timepoints, adhesion scores were highest in the untreated group. At both timepoints, extraneural collagen deposition was significantly higher in the untreated group than in the sham and the HA-SIS groups. At 26 weeks, CD68-positive macrophages were significantly higher in the untreated group than in the HA-SIS group. No significant differences were noted across groups for intraneural collagen-to-cell ratio and blood vessel count.

**Conclusions:**

These results demonstrate that nerve wrapping with HA-SIS in an injured muscle bed reduces external nerve adhesions, extraneural collagen deposition, and long-term inflammation (CD68-positive macrophages) compared with the results of unwrapped nerves.

**Clinical relevance:**

This level five study examines the impact of a hostile environment on nerve health, as well as the influence of a nerve protection device on diminishing its detrimental effects.

Entrapment neuropathy may be an acute or chronic condition caused by trauma or repetitive motion injuries. Independent of the cause, this condition potentially results in pain, paresthesia, and paresis for the patient. Nerve entrapments commonly occur in fibro-osseous tunnels such as the carpal, cubital, or tarsal tunnel^1^ and mandibular nerve.^2^ However, they can also occur between soft tissue planes including the greater, lesser, and third occipital nerves^3^; the periprostatic space after biopsies or other prior prostate procedures^4^; and nonspecific scar neuropathy.^5^ If conservative treatments fail, surgical treatment of the entrapment neuropathy can be performed by external neurolysis, such that the nerve is freed from compressing structures and adhesions, allowing for nerve movement through the range of motion.^6^^,^^7^ Surgical nerve decompression alone often results in favorable outcomes; however, the recurrence of secondary peripheral nerve adhesions occurs in a small subset of patients.^8^ The treatment of nerve entrapments remains a difficult task, as nerve trauma with subsequent swelling increases the likelihood of focal ischemia and scarring, leading to adhesions.^9^^,^^10^ These nerve entrapments may also be exacerbated depending on the injury mechanism, where cautery leads to more drastic scarring within and around the nerve.^10^ Treating these recurrent neuropathies may include surgically decompressing the nerve combined with a method to prevent recurrent nerve scarring, such as a fat flap or application of a nerve wrap.^11^ The application of a nerve wrap has shown promising results to limit nerve scarring and adhesions.^12^

Histological evaluation of wrapped and unwrapped nerves has shown notable extraneural scar tissue formation and nerve degeneration in unwrapped nerves, whereas wrapped nerves have shown improved functional nerve recovery by acting as a barrier to scar tissue infiltration from the surrounding soft tissues.^13^ One promising approach to protect the injured nerve involves using off-the-shelf nerve wraps.^14^ These nerve wraps create a physical barrier around the nerve that promotes nerve gliding, allows for diffusion of nutrients, prevents axonal escape, and reduces scar tissue formation.^15^ Various materials have been used for nerve wrapping including autografts (eg, vein wraps), synthetic materials (eg, polycaprolactone), and natural biomaterials (eg, type 1 bovine collagen, porcine small intestine submucosa, amniotic membrane).^7^^,^^15^ An ideal nerve barrier should have minimal inflammation or chance of rejection, porosity that facilitates nutrient diffusion, minimal scar-induced ischemia, ability to support nerve gliding, and minimal or no donor site morbidity.^15^ Hyaluronate gels can be bonded directly to underlying substrates to reduce intraneural and extraneural scar tissue formation and provide mechanical integrity with a stronger underlying material.^16^^,^^17^ Furthermore, wrapping a nerve with porcine small intestine submucosa (pSIS) has previously shown success reducing adhesions in a preclinical model^18^; therefore, we hypothesized that coating pSIS with hyaluronate would provide a physical barrier inhibiting the migration of inflammatory cells and reduce the likelihood of intraneural fibrosis and extraneural adhesions. To test this hypothesis, we reproduced a rat sciatic nerve model using a heat coagulation-induced injury to the muscle bed surrounding the nerve to generate a hostile environment.^10^ Nerves in the treatment group were wrapped with hyalurnoate/alginate-coated pSIS nerve wrap.

## Materials and Methods

Sixty male Lewis rats aged 8–10 weeks and weighing 200–300 g were used for the study. All surgical procedures and animal care conformed to the National Institutes of Health guidelines, were performed in accordance with the standard of care practices at the USF Morsani College of Medicine and Heart Institute, and were approved by the University of South Florida Institutional Animal Care and Use Committee. Animals were randomly assigned to one of three groups: sham (n = 20), untreated (n = 20), or HA-SIS (n = 20) as described below. Ten animals in each group were terminated at both 6 weeks and 26 weeks. This sample size was chosen based on pilot data that indicated an *n* value of 10 would provide adequate power to show the difference between groups at *P* < 0.05.

### Surgical procedures

All surgical procedures were performed using a surgical microscope (Leica model F12, Leica Microsystems). In all rats, the sciatic nerve in the right hindlimb was isolated using standard surgical procedures. In the sham group, the sciatic nerve was exposed and neurolyzed. Silicone and saline-soaked alginate foam (#A6209; Simpurity Foam, Safe N Simple) was then applied, manipulated, and removed to simulate other groups’ handling. The surgical site was then immediately closed using standard surgical technique. In the untreated and HA-SIS groups, the soft tissue surrounding the nerve was cauterized with a bipolar coagulator to induce tissue disruption and create a scarred wound bed, as previously described.^10^ The tips of the bipolar coagulator forceps were fixed 5 mm apart using a 3-dimensional printed holder and held at an approximately 45° angle to the soft tissue. The tissue was cauterized with the bipolar coagulator for 3 seconds at 35 W, then the forceps were advanced along the edge of the tissue with consistent spacing between coagulated areas until a muscle injury measuring 10 × 20 mm was created. During the cauterization process, the nerve was isolated and protected using minimum force with an outer layer of surgical silicone and an inner layer of saline-soaked wound dressing (#A6209; Simpurity Foam, Safe N Simple) to prevent nerve damage. The area of cauterization began approximately 5–7 mm distal to the iliac fascia and was positioned so that the sciatic nerve was centered within the 10 × 20 mm disrupted wound bed, 10 mm proximal to distal, and 20 mm superior to inferior. No additional treatment was applied in the untreated group, and the surgical site was closed using standard surgical techniques. In the HA-SIS group, a 10 × 15 mm Axoguard HA+ Nerve Protector (Axogen Corporation) was placed around the nerve after the injury was created, ensuring that the wrap was centered within the injured muscle bed with the 15 mm length in the proximal to distal direction. Each nerve wrap was secured to itself along the longitudinal slit with two 8-0 sutures placed approximately 2 mm from the open end of the wrap. To prevent overtightening, a pair of tweezers were carefully inserted during suture placement, with the two prongs aligned radially between the nerve and the wrap. This technique helped maintain an appropriate gap and avoid excessive compression of the nerve. Each surgical site was closed using standard surgical techniques. Animals recovered for 6 weeks or 26 weeks and were then killed humanely.

Gastrocnemius muscles were collected from both legs of each animal. Muscle weight was recorded for the gastrocnemius muscle of the injured leg, which was expressed as a percentage of the gastrocnemius muscle weight of the contralateral healthy leg, termed percent gastrocnemius muscle weight. Adhesion assessments were conducted using a previously established scale.^10^^,^^18^ After opening the muscle plane, the evaluator only manipulated the upper plane to preserve underlying tissues for histological evaluation; the fibrotic tissue between the nerve and surrounding areas for nerve mobility, tissue transparency, ease of separation, and coverage extent was assessed as outlined in the Table 1.

**Table 1:** Nerve Adhesion Scoring Was Performed Before the Sample Explant

Category	Evaluation Criteria and Description		Scores
Nerve immobility, tested by pulling on the nerve perpendicular and longitudinal to the nerve axis	Nerve moves freely, as compared with the contralateral healthy nerve		0
	Nerve movement is restricted by scar tissue, pulling the surrounding tissues		1
	Nerve is completely immobilized by the scar tissue		2
Adhesion tissue quality (Circle all that apply, select the highest score, and do not add scores; the highest score is selected and recorded, max = 4)	None, similar to a healthy contralateral nerve		0
	Appearance	Transparent	1
		Translucent	2
		Opaque	3
	Vascularization	Avascular	1
		Capillaries present	3
		Large vessels present	4
Adhesion tenacity (max = 3)	None, similar to a healthy contralateral nerve		0
	Adhesions fall apart with blunt dissection (applying gentle traction using forceps)		1
	Adhesions tear with significant traction (using forceps to pull two ends but do not need cutting)		2
	Adhesions require sharp transection (require transection using scissors)		3
Extent of site involvement for adhesions	None, similar to a healthy contralateral nerve		0
	<50% of the exposed nerve		2
	>50% of the exposed nerve		4
Total adhesion score: a cumulative of nerve immobility, adhesion quality, tenacity, and extent of site involvement for adhesions	Add the highest score from each assessment criterion above (nerve immobility, adhesion quality, tenacity, and extent of site involvement for adhesions)		0–13

### Histology

Samples of the sciatic nerve and underlying muscle bed were recovered from approximately 10 mm proximal to the muscle bed injury site to approximately 10 mm distal to the muscle bed injury site. All samples were marked with a tissue marker or suture on the proximal nerve stump. The tissue was fixed in 10% neutral-buffered formalin for 48 hours. Samples were embedded in paraffin blocks. Serial sections were cut 5-μm thick; one section was used for each type of histology staining.

Nerve sections were stained with Masson’s trichrome (MT) to evaluate extraneural collagen and collagen-to-cell ratio, anti-CD68 (CD68) to evaluate macrophage cells, and anti-α smooth muscle actin (αSMA) to evaluate vascularization. Immunohistochemistry slides were prepared after sectioning, as follows: Heat-mediated antigen-retrieval was performed by placing the slides in sodium citrate buffer (pH 6; Abcam). Peroxidase activity and nonspecific protein binding were quenched using Bloxall (Abcam) and 10% normal goat serum with 1% bovine serum albumin in tris-buffered saline, respectively. Slides were then stained with antibodies to CD68 (ab125212, 1:500 dilution, Abcam) or α-smooth muscle actin (αSMA, ab124964, 1:1000, Abcam).

For CD68 staining, incubation with goat antirabbit immunoglobulin G heavy and light chains followed by horseradish peroxidase-conjugated secondary antibody (ab205718, 1:1000 dilution, Abcam) and then exposure to diaminobenzidine (DAB, ab64238, Abcam) to visualize immunoreactivity. Sections were then counterstained in hematoxylin, dehydrated, and cover-slipped. For αSMA staining, incubation of rabbit anti-αSMA antibody was followed by a fluorescent secondary antibody (#PIA3274 for Alexa Fluor Plus 594, Fisher Scientific; 1:400 dilution) and counterstaining of cell nuclei with 4',6-diamidino-2-phenylindole (DAPI) (#62248, Thermo Scientific; 1:1000). The sections were then dehydrated and cover-slipped.

Sections were scanned using a Zeiss Axio Scan Z1 automated slide scanner (Carl Zeiss Spectroscopy GmbH). Slides stained with MT and CD68 were scanned using the bright-field light source at 200× magnification. Slides stained with αSMA were scanned using the fluorescent light source at 400× magnification. The Zeiss Axio Scan Z1 automated slide scanner rendered a single image for each histology section. Images were collected for the entire sample cross-section. Histologic quantification was performed using ImageJ (National Institutes of Health).

Images of MT-stained slides were used to calculate extraneural collagen area and collagen-to-cell ratio. Extraneural collagen and collagen-to-cell ratio were quantified by adjusting the hue, saturation, and brightness levels of MT-stained slide images to capture the relevant blue area that corresponded to the collagen. Using the same function, the manual color threshold was adjusted to select the pink areas corresponding to cellular cytoplasm. Collagen-to-cell ratio was obtained by dividing the intraneural collagen area by the cellular area. ImageJ then generated a spreadsheet for each analyzed image that highlighted the area for each particle. For the extraneural collagen analysis, we applied the same thresholding technique as described above for the blue pixels encircling the nerve fascicles. The collagen was quantified and expressed as area in square micrometers.

Immunohistochemistry slides were used to quantify CD68-positive (CD68^+^) macrophages and blood vessels. Quantification of CD68^+^ macrophages used positively stained area measurements from images manually thresholded for brown areas corresponding to positive cellular staining. The pixel area was measured within the cross-sectional area of the nerve and recorded as the percent of CD68^+^ macrophages in the total nerve area. Immunofluorescence staining for smooth muscle actin was employed to identify vascular components within the nerve. Blood vessels were enumerated within the nerve’s cross-sectional areas using the cell counter function of ImageJ, and the results were noted as the number of vessels within the nerve.

### Statistical analysis

Given the high variability inherent in animal data, statistical analysis was performed with Kruskal-Wallis tests. The main effects were considered significantly different when the *P* value was less than or equal to 0.05. Results are reported as mean ± standard deviation (SD) and 95% confidence interval (95% CI).

## Results

**Figure 1:**
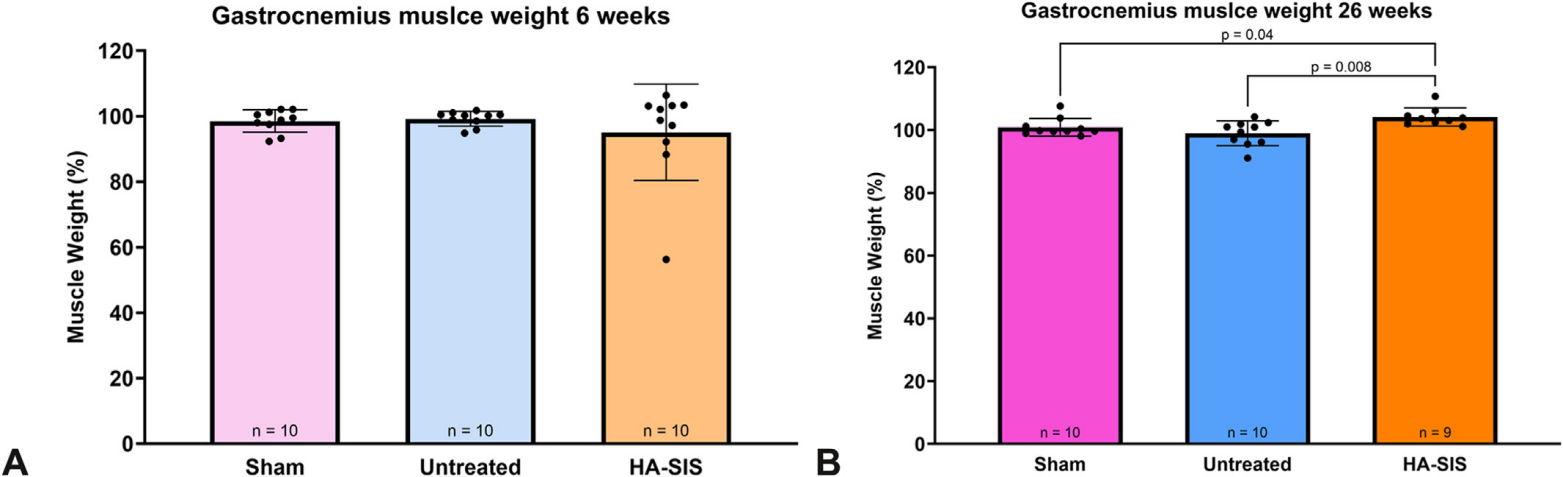
Gastrocnemius wet weight of the surgical leg at **A** 6 weeks and **B** 26 weeks, expressed as a percent of the gastrocnemius muscle wet weight of the non-surgical contralateral leg. Graphical data presented as mean ± SD.

All results were collected 6 weeks or 26 weeks after surgery. No adverse reactions were noted throughout the course of the study. At 6 weeks, gastrocnemius muscle weight showed no significant differences between groups (Fig. 1A and Table 2). At 26 weeks, the HA-SIS group had a significantly higher percentage of muscle weight than both the sham and the untreated groups, *P* = .04 and *P* = .008, respectively (Fig. 1B).

**Table 2:** Gastrocnemius Muscle Wet Weight of the Surgical Leg, Expressed as a Percentage of the Gastrocnemius Muscle Wet Weight of the Contralateral Healthy Leg

Timepoint	6 Wk		26 Wk	
Group	Gastrocnemius Muscle Weight (%) Mean ± SD	Gastrocnemius Muscle Weight (%) 95% CI	Gastrocnemius Muscle Weight (%) Mean ± SD	Gastrocnemius Muscle Weight (%) 95% CI
Sham	98.5 ± 3.4	96.1–101.0	100.9 ± 2.8	98.8–102.9
Untreated	99.2 ± 2.3	97.6–100.8	98.9 ± 4.0	96.1–101.8
HA-SIS	95.1 ± 14.7	84.6–105.6	104.1 ± 2.9	101.9–106.3

**Figure 2:**
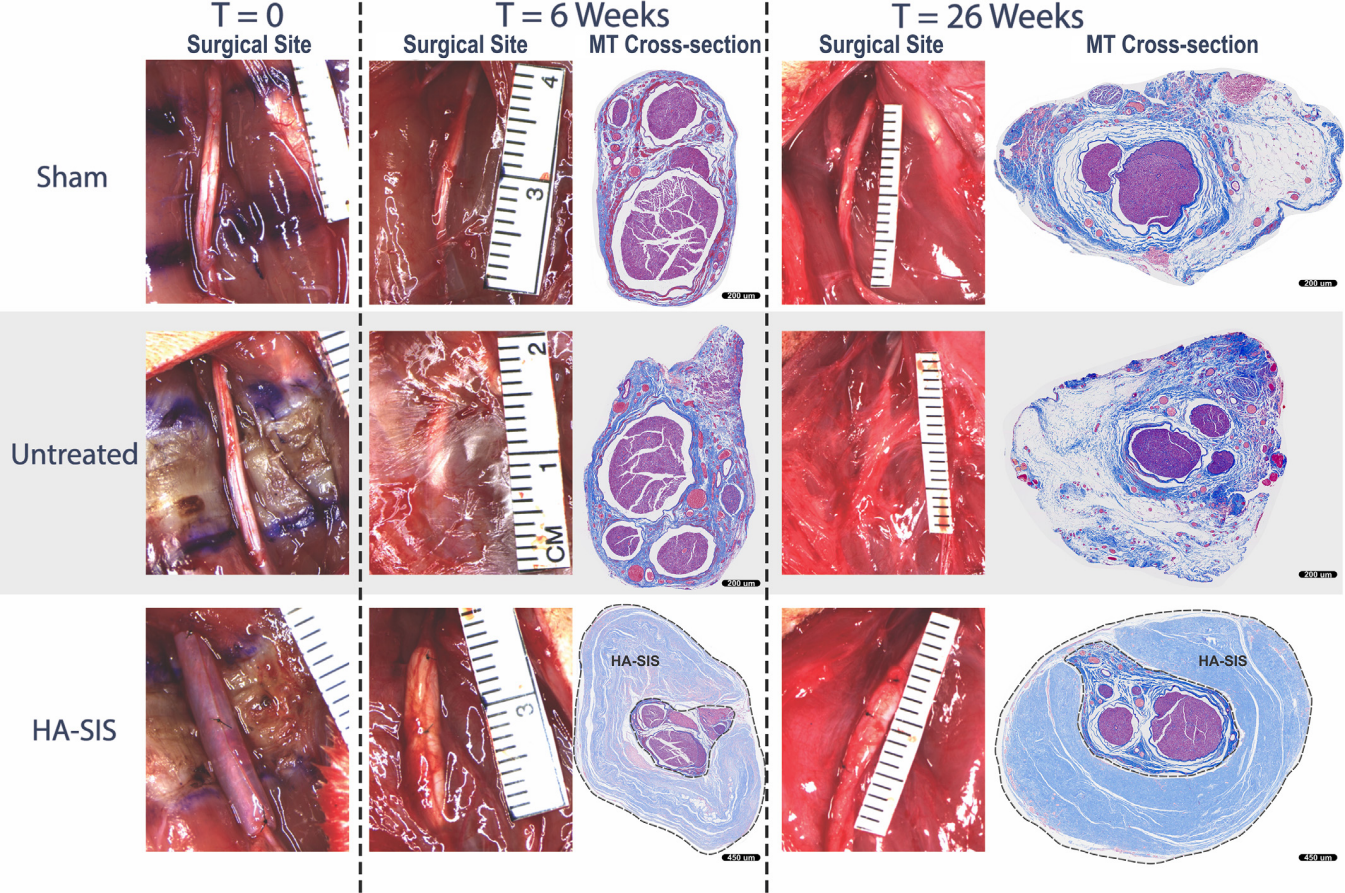
Representative images of the sciatic nerve immediately after surgery and before closing the surgical site (T = 0) in sham procedure (top row), untreated (middle row), and HA-SIS group (bottom row). Representative images of Masson's Tricrhome-stained histology cross-sections (scale bars at 250 μm in the sham and untreated groups, scale bars at 450 μm in the HA-SIS group) of the sciatic nerve at 6 weeks (second column) and 26 weeks (third column).

During gross pathology evaluation at 6 weeks and 26 weeks, a thick fibrotic connective tissue was observed covering the nerve in the untreated group, requiring traction with blunt dissection. The connective tissue covering the nerves of the untreated group was also notably thicker than in the sham or the HA-SIS groups, which was especially noticeable at 26 weeks. The connective tissue in the HA-SIS group was transparent, smooth, and easier to remove from the nerve. These findings were also apparent in the MT-stained histology cross-sections, which showed a connective tissue layer surrounding the nerve in all groups (Fig. 2).

**Figure 3:**
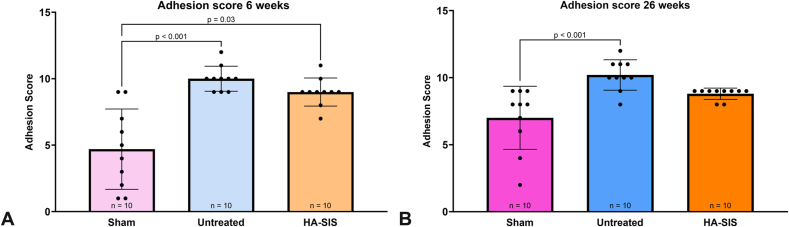
Adhesion scores at **A** 6 weeks and **B** 26 weeks. Graphical data presented as mean ± SD.

**Figure 4:**
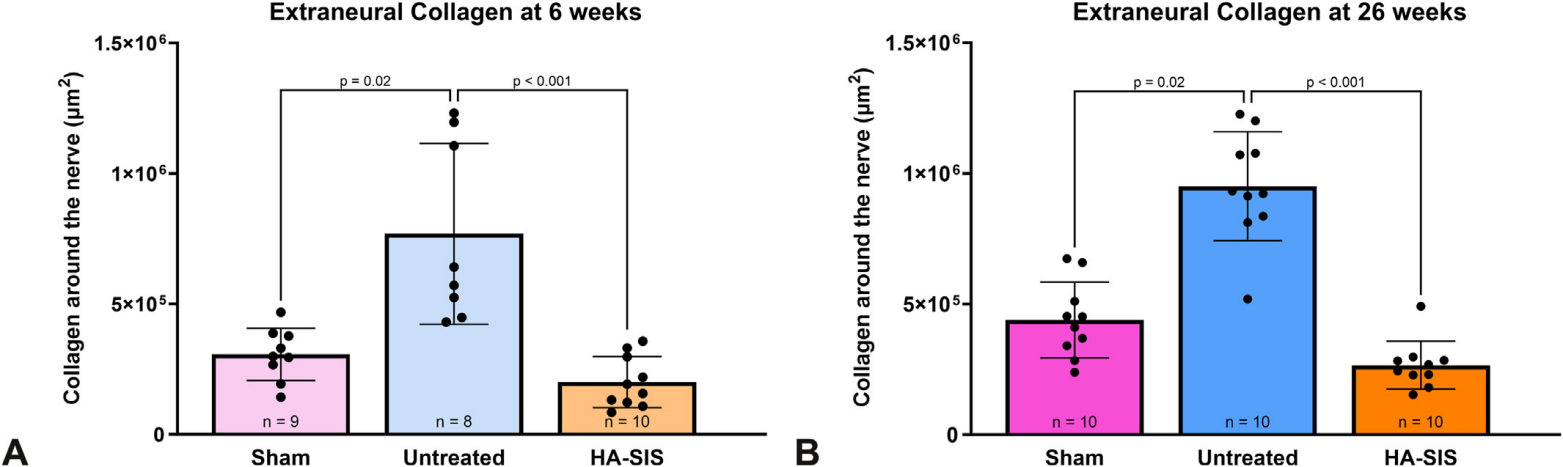
Extraneural collagen at **A** 6 weeks and **B** 26 weeks. Graphical data presented as mean ± SD.

At 6 weeks, the sham exhibited a significantly lower adhesion score than either the untreated or HA-SIS groups, *P* < .001 and *P* = .03, respectively (Fig. 3A and Table 3). At 26 weeks, there were no significant differences in adhesion score between the HA-SIS group and the sham group, but the adhesion score of the untreated group remained significantly higher than that of the sham group, *P* < .001 (Fig. 3B). Additional evaluations of collagen surrounding the nerve were performed using quantitative measures of MT-stained histology slides, termed extraneural collagen. At both time points, the sham and HA-SIS groups had significantly less extraneural collagen than the untreated group at 6 weeks (Fig. 4A) and 26 weeks (Fig. 4B), *P* = .02 and *P* < .001, respectively.

**Table 3:** Adhesion Scores and Extraneural Collagen

Timepoint	6 Wk		26 Wk	
Group	Adhesion Score Mean ± SD	Adhesion Score 95% CI	Adhesion Score Mean ± SD	Adhesion Score 95% CI
Sham	4.7 ± 3.0	2.5–6.9	7.0 ± 2.4	8.5–9.1
Untreated	10.0 ± 0.9	9.33–10.67	10.2 ± 1.1	9.4–11.0
HA-SIS	9.0 ± 1.1	8.23–9.75	8.8 ± 0.4	8.5–9.1

	Extraneural Collagen (μm^2^) Mean ± SD	Extraneural Collagen (μm^2^) 95% CI	Extraneural Collagen (μm^2^) Mean ± SD	Extraneural Collagen (μm^2^) 95% CI
Sham	3.060 × 10^5^ ± 9.98 × 10^4^	2.30 × 10^5^ − 3.83 × 10^5^	4.39 × 10^5^ ± 1.45 × 10^5^	3.36 × 10^5^ − 5.42 × 10^5^
Untreated	7.69 × 10^5^ ± 3.47 × 10^5^	4.78 × 10^5^ − 1.06 × 10^6^	9.51 × 10^5^ ± 2.08 × 10^5^	8.02 × 10^5^ − 1.10E + 0^6^
HA-SIS	2.0 × 10^5^ ± 9.78 × 10^4^	1.30 × 10^5^ − 2.70 × 10^5^	2.66 × 10^5^ ± 9.16 × 10^4^	2.00 × 10^5^ − 3.32 × 10^5^

**Figure 5:**
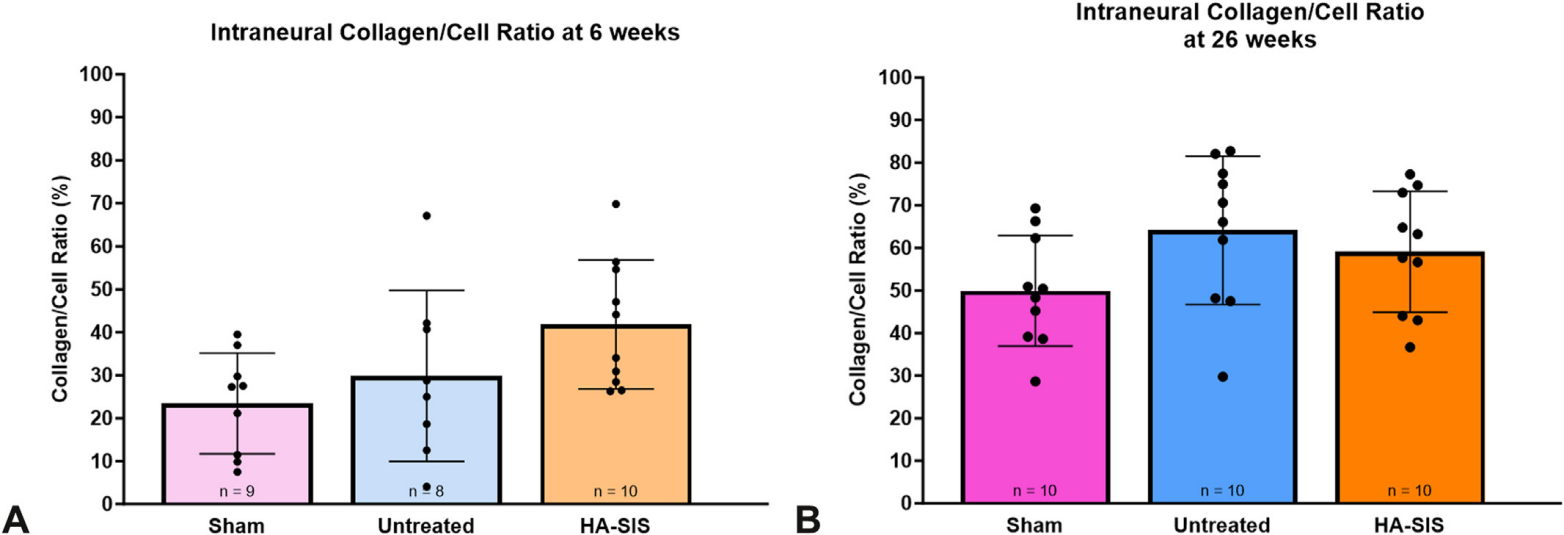
Intraneural collagen-to-cell ratio at **A** 6 weeks and **B** 26 weeks. Graphical data presented as mean ± SD.

**Figure 6:**
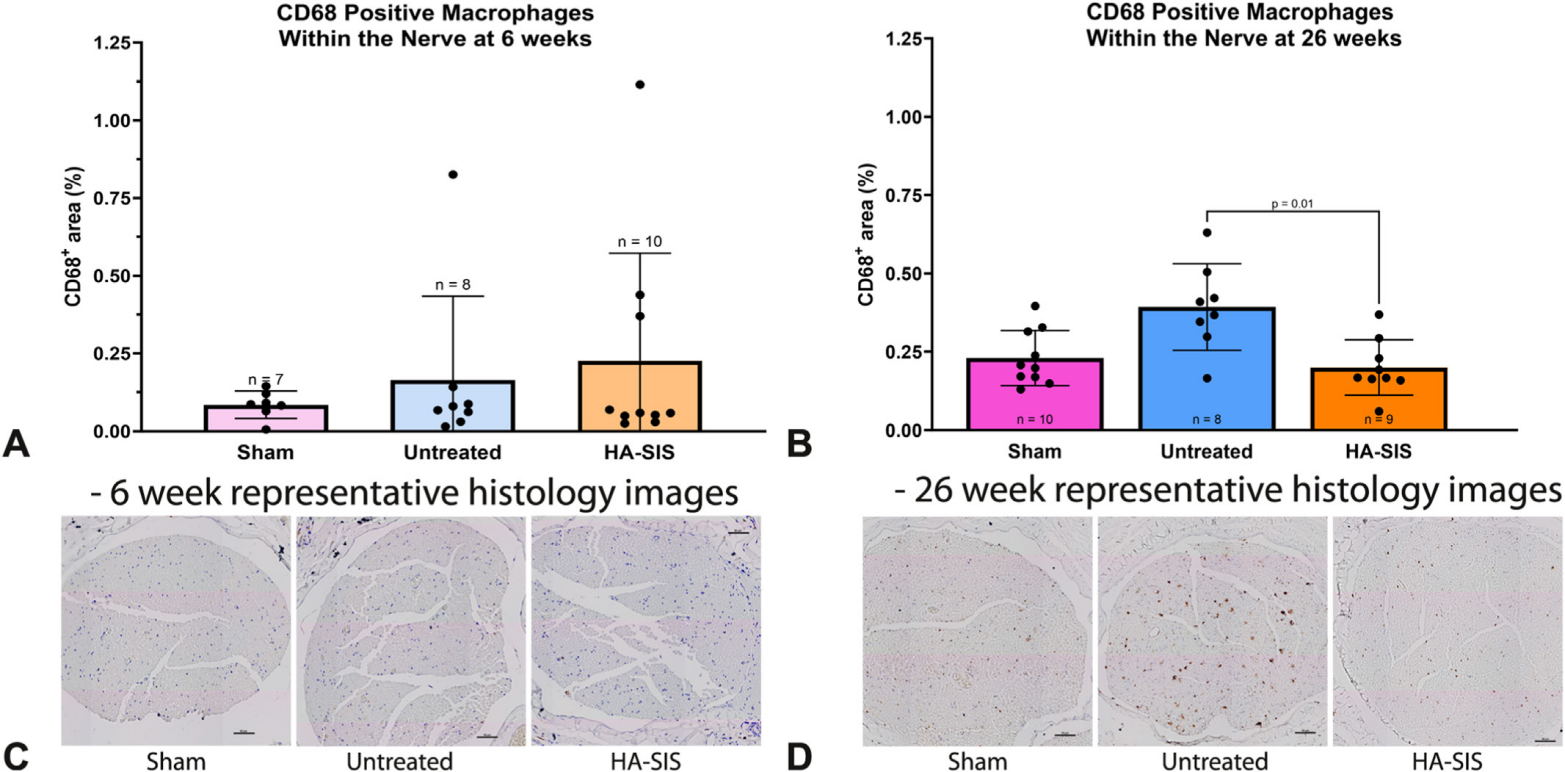
CD68^+^ macrophage area within the nerve at **A** 6 weeks and **B** 26 weeks. Graphical data presented as mean ± SD: **C** representative CD68-stained histology images of each study group at 6 weeks, scale bar at 50 μm, and **D** representative CD68-stained histology images of each study group at 26 weeks, scale bar at 50 μm.

**Figure 7:**
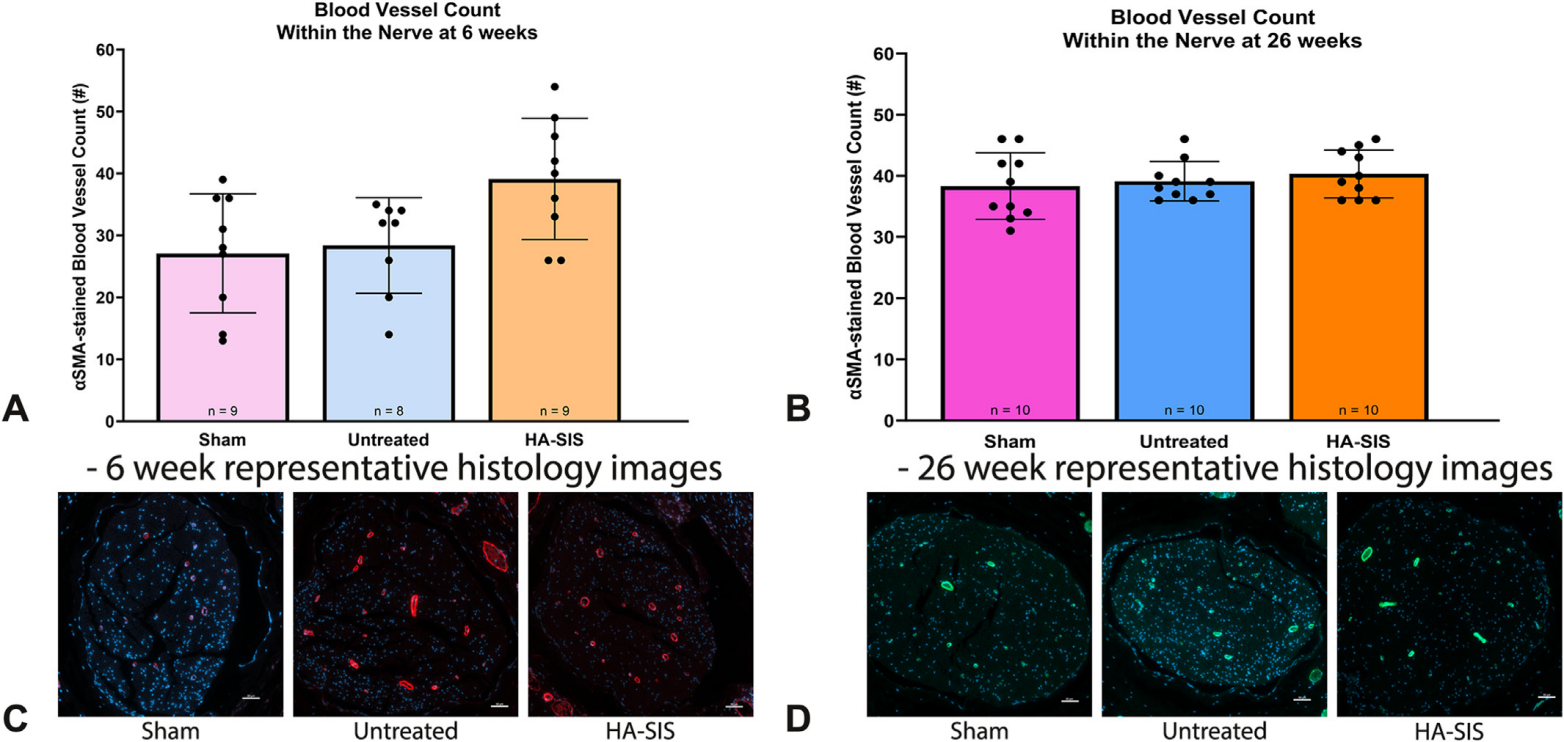
Blood vessel count within the nerve at **A** 6 weeks and **B** 26 weeks. **C** Representative αSMA-stained histology images, scale bars at 50 μm, of each study group at 6 weeks. **D** Representative αSMA-stained histology images of each study group at 26 weeks, scale bars at 50 μm.

There were no statistical differences between groups with respect to intraneural collagen-to-cell ratio at 6 weeks (Fig. 5A and Table 4) and 26 weeks (Fig. 5B). All groups at 6 weeks had comparable CD68^+^ areas (Fig. 6A, C, and Table 5). This demonstrated similar early inflammation patterns within the nerve’s fascicular area across the groups. However, at 26 weeks, a larger percentage area of CD68^+^ macrophages was noted in the untreated group compared with HA-SIS, *P* = 0.01 (Fig. 6B, D). The sham group was not significantly different from either group. At 6 weeks, the HA-SIS group had more blood vessels, but there were no significant differences at either timepoint (Fig. 7A–D and Table 6).

**Table 4:** Collagen-to-Cell Ratio

Timepoint	6 Wk		26 Wk	
Group	Collagen-to-Cell Ratio (%) Mean ± SD	Collagen-to-Cell Ratio (%) 95% CI	Collagen-to-Cell Ratio (%) Mean ± SD	Collagen-to-Cell Ratio (%) 95% CI
Sham	23.4 ± 11.7	14.4–32.5	49.9 ± 13.0	40.6–59.2
Untreated	29.9 ± 19.9	13.2–46.5	64.1 ± 17.4	51.7–76.6
HA-SIS	41.8 ± 15.0	31.1–52.5	59.1 ± 14.2	49.0–69.3

**Table 5:** CD68^+^ Macrophage Area

Timepoint	6 Wk		26 Wk	
Group	CD68^+^ Macrophage Area (%) Mean ± SD	CD68^+^ Macrophage Area (%) 95% CI	CD68^+^ Macrophage Area (%) Mean ± SD	CD68^+^ Macrophage Area (%) 95% CI
Sham	0.09 ± 0.04	0.04–0.13	0.23 ± 0.09	0.17 – 0.29
Untreated	0.16 ± 0.27	−0.06 to 0.39	0.39 ± 0.14	0.28 – 0.51
HA-SIS	0.23 ± 0.35	−0.02 to 0.47	0.20 ± 0.09	0.13 – 0.27

**Table 6:** Blood Vessel Count Within the Nerve

Timepoint	6 Wk		26 Wk	
Group	Blood Vessel Count (#) Mean ± SD	Blood Vessel Count (#) 95% CI	Blood Vessel Count (#) Mean ± SD	Blood Vessel Count (#) 95% CI
Sham	27.1 ± 9.6	19.7–34.5	38.3 ± 5.5	34.4–42.2
Untreated	28.4 ± 7.7	21.9–34.8	39.1 ± 3.2	36.8–41.4
HA-SIS	39.1 ± 9.8	31.6–46.6	40.3 ± 3.9	37.5–43.1

## Discussion

In a hostile nerve environment, various materials have been employed as nerve wraps to form protective barriers around nerves, which may minimize unwanted adhesions and preserve nerve integrity. Common materials for peripheral nerve wraps include vein allografts, amniotic membranes, bovine type 1 collagen, pSIS, and synthetic materials.^14^^,^^19^ A systematic review of nerve wrapping in rat models found that the application of a nerve wrap to prevent nerve adhesions is promising and may depend on both technique and material.^12^ Nerve wrapping material should have properties well-suited for application around a nerve including biocompatibility; biomechanical integrity through regeneration while also being flexible enough to not cause tissue irritation; absorbability throughout the remodeling process; semi-permeable to act as a barrier to cell infiltration from surrounding soft tissues and contain neurotropic milieu while also allowing for oxygen and nutrient diffusion; the ability to allow nerve mobility; and overall support nerve regeneration.^15^^,^^19^^,^^20^ Ultimately, these biomaterial properties provide a barrier to prevent attachments between the nerve and surrounding soft tissue while also supporting nerve regeneration.^20^ Although current literature does not provide a clear conclusion on ideal material for nerve wrapping, tissue-derived materials reduce scar density and nerve stiffness.^12^ We hypothesized that using an HA-coated nerve wrap would provide a physical barrier inhibiting the migration of the inflammatory cells and reduce the likelihood of intraneural fibrosis and extraneural attachments.

Gross pathology in our study showed a thick fibrotic connective tissue covering the nerves of the untreated group, which was notably thicker than the connective tissue in the sham or HA-SIS groups. Although the HA-SIS group also showed a connective tissue covering the nerve, the tissue was transparent and easier to separate from the nerve. Macroscopically, the 6-week semi-quantitative adhesion scores were higher in both the HA-SIS and untreated groups compared with the sham group. Interestingly, only the untreated group maintained this significant difference through the 26-week period. This suggests that while HA-SIS-treated and untreated groups initially display increased adhesion, the untreated group demonstrates a more sustained impact.

Extraneural collagen was significantly higher in the untreated group than in the sham or HA-SIS groups at both timepoints, indicating less formation of attachments. These results parallel prior literature showing that protective wraps reduced extraneural attachments.^21^^,^^22^ Although hyaluronate and alginate lack the mechanical integrity to serve as a nerve wrap, coating a mechanically stable nerve wrap like pSIS may further reduce scar formation beyond a nerve wrap alone.^17^

The presence of macrophages in a regenerating nerve has been linked to ongoing Wallerian degeneration, as macrophages remove myelin and axonal debris.^23^ At 6 weeks, all groups showed similar concentration of CD68^+^ macrophages, consistent with early inflammation in Wallerian degeneration, as the transected sciatic nerve has shown detectable macrophages for up to 8 weeks.^24^ Long-term presence of macrophages may be indicative of unresolved Wallerian degeneration, chronic inflammation, and impaired regeneration.^23^^,^^25^ At 26 weeks, the percentage area of CD68^+^ macrophages was highest in the untreated group and lowest in the HA-SIS and sham groups. Notably, the untreated group showed significantly more CD68^+^ macrophages than the HA-SIS group at 26 weeks. These findings show that wrapping a nerve with HA-SIS functions as a barrier for inflammatory cells in a hostile wound environment while allowing for the resolution of Wallerian degeneration. The collagen-to-cell ratio at 26 weeks was highest in the untreated group, indicating slightly more intraneural fibrosis at this long-term timepoint. Previous evaluation using collagen-to-cell ratio showed trending differences, but not significant differences, between injury of the soft tissue with a bipolar coagulator and sham injury.^10^ This evaluation may be inadequate to detect differences between groups with respect to intraneural fibrosis. The elevated adhesion scores and extraneural collagen in the untreated group are evidence of fibrosis and extraneural adhesions in the untreated group. The presence of extraneural adhesion may also have contributed to the neural deficits leading to significantly lower gastrocnemius wet muscle weight in the sham and untreated groups compared with the HA-SIS group. Although muscle weight showed significant differences, it is possible that the differences in weight may be attributable to the variables in the collection of the gastrocnemius muscle, as the mean gastrocnemius muscle weight ratio ranged between 95.1% and 104.1%.

The accumulation of collagen around the nerves of the untreated group is consistent with scar tissue formation, which may present clinically as a compressive neuropathy. Although this study is inherently limited to evaluating the use of HA-SIS for clinical outcomes because of the nature of preclinical animal study evaluations, the data suggest potential benefits of using a nerve wrap in a hostile wound environment. Revision surgery for carpal and cubital tunnel syndrome often has incomplete ligament release or areas of newly formed spot attachments or circumferential encasement causing compression or tethering to the surrounding soft tissues.^15^ Nerve wraps have been shown to improve outcomes with no implant-related complications in patients who underwent revision cubital tunnel surgery with the application of a pSIS wrap.^14^ The addition of HA hydrogel to SIS creates a multilayer barrier with both a short-term resorbable layer (HA hydrogel) and a longer-term remodeling layer (SIS), which may further enhance the protective properties of the nerve wrap. The relative decrease in adhesions, extraneural fibrosis, and intraneural inflammatory cells observed in this study suggests potential utility as a nerve wrap. Subsequent clinical analyses may be performed to understand the clinical benefits of applying HA-SIS nerve wrap to a nerve in a hostile wound bed.

## Conflicts of Interest

Dr Alsmadi is an Axogen employee and shareholder. Dr Deister is an Axogen employee and shareholder. Dr Evans is an Axogen consultant. Dr Ghanem is on the Axogen advisory board. Dr Mercer is an advisor and speaker for Axogen. Dr Smetana is a consultant for Axogen and serves on the advisory board. This research was funded by and conducted with the assistance of Axogen Corporation. Bias in this study was controlled by statistical evaluation of histology sections prepared from nerve samples.
